# Evaluation of a Type 2 diabetes risk reduction online program for women with recent gestational diabetes: a randomised trial

**DOI:** 10.1186/s12966-022-01275-3

**Published:** 2022-03-28

**Authors:** Rachael Taylor, Megan E. Rollo, Jennifer N. Baldwin, Melinda Hutchesson, Elroy J. Aguiar, Katie Wynne, Ashley Young, Robin Callister, Clare E. Collins

**Affiliations:** 1 School of Health Sciences, College of Health, Medicine and Wellbeing, University of Newcastle, Callaghan, New South Wales (NSW) Australia; 2 Priority Research Centre for Physical Activity and Nutrition, University of Newcastle, Callaghan, NSW Australia; 3 Hunter Medical Research Institute, New Lambton Heights, NSW 2305 Australia; 4 Department of Kinesiology, College of Education, The University of Alabama, Tuscaloosa, USA; 5 School of Medicine and Public Health, College of Health, Medicine and Wellbeing, University of Newcastle, Callaghan, NSW Australia; 6 Department of Diabetes & Endocrinology, John Hunter Hospital, Hunter New England Health District, New Lambton, NSW 2305 Australia; 7 Clinical Services, Nursing and Midwifery, Hunter New England Local Health District, Wallsend, NSW Australia; 8 School of Biomedical Sciences and Pharmacy, College of Health, Medicine and Wellbeing, University of Newcastle, Newcastle, Callaghan Australia; 9 Level 3 Advanced Technology Centre, Ring Road, Priority Research Centre in Physical Activity and Nutrition, The University of Newcastle, Callaghan, New South Wales 2308 Australia

**Keywords:** Gestational diabetes, Type 2 diabetes, Prevention, Weight loss, Diet, Exercise

## Abstract

**Background:**

To evaluate the preliminary efficacy, feasibility and acceptability of the 3-month Body Balance Beyond (BBB) online program among Australian women with overweight/obesity and recent gestational diabetes mellitus.

**Methods:**

Women were  randomised into either: 1) High Personalisation (HP) (access to ‘BBB’ website, video coaching sessions, text message support); 2) Medium Personalisation (MP) (website and text message support); or 3) Low Personalisation (LP) (website only). Generalised linear mixed models were used to evaluate preliminary efficacy, weight, diet quality, physical activity levels, self-efficacy and quality of life (QoL) at baseline and 3-months. Feasibility was assessed by recruitment and retention metrics and acceptability determined via online process evaluation survey at 3-months.

**Results:**

Eighty three women were randomised, with 76 completing the study. Self-efficacy scores showed significant improvements in confidence to resist eating in a variety of situations from baseline to 3-months in HP compared to MP and LP groups (*P*=.03). The difference in mean QoL scores favoured the HP compared to MP and LP groups (*P*=.03). Half of the women (HP *n*=17[81%], MP *n*=12[75%], LP *n*=9[56%]) lost weight at 3-months. No significant group-by-time effect were reported for other outcomes. Two-thirds of women in the HP group were satisfied with the program overall and 86% would recommend it to others, compared with 25% and 44% in the MP group, and 14% and 36% in the LP group, respectively.

**Conclusions:**

Video coaching sessions were associated with improvements in QoL scores and self-efficacy, however further refinement of the BBB website and text messages support could improve program acceptability.

**Trial registration:**

Australian New Zealand Clinical Trials Registry (ANZCTR): ACTRN12619000162112, registered 5 February 2019.

**Supplementary Information:**

The online version contains supplementary material available at 10.1186/s12966-022-01275-3.

## Background

Worldwide, the burden due to type 2 diabetes mellitus (T2DM) on individuals, health systems and national economies continues to increase. In 2019, the International Diabetes Federation estimated that 463 million (9.3% of the population) adults aged 20–79 years were living with diabetes, with over 90% of cases due to T2DM [1, 2]. Prevalence of T2DM is projected to increase to 700 million (10.9%) adults by 2045 [1, 2].

The incidence of gestational diabetes mellitus (GDM) is a risk factor unique to women for future development of T2DM [3]. GDM is defined as any degree of glucose intolerance that occurs during any stage of pregnancy [4]. Globally, one in six births are affected by GDM [1, 2]. In Australia, GDM prevalence has risen from 5 to 15% of pregnancies in the past 2 decades [5]. A recent meta-analysis of 30 cohort studies, including more than 2.6 million women, indicated that GDM was associated with a 7.76 fold (95% confidence intervals: 5.10-11.81) increased risk of developing T2DM with the highest level of risk occurring within 3-6 years after GDM in women under 40 years of age [3]. These findings indicate that the first 3 years following GDM is a critical window for interventions targeting T2DM prevention in this high-risk population.

Lifestyle interventions are effective for T2DM prevention in middle-aged adults, with the risk of diabetes being reduced by 46-58% [6, 7]. However, the efficacy of these interventions in women with a history of GDM is unclear. A meta-analysis of 8 randomised controlled trials (RCTs) found that lifestyle interventions, mostly delivered via face-to-face consultations, were associated with a 25% lower risk of diabetes in women with a history of GDM [8]. These preliminary findings suggest that the effect of lifestyle interventions on diabetes risk is smaller in postpartum women compared to middle-aged adults.

Emerging evidence indicates that personalising lifestyle interventions to the characteristics of individuals (e.g. beliefs/values, health behaviours, disease risk markers) may be a critical factor for faciliating improvements in health behaviours. Findings from a systematic review indicated that 8 of 11 randomised control trials (*n*=3869 adults) reported improvements in dietary intake in adults who have received personalised nutrition advice compared with the control group receiving generalised dietary advice [9]. However, a scoping review of personalised physical activity interventions highlighted mixed findings [10], which the reviewers attributed to substantial variation in study sample sizes and hence study power, difference in intervention intensity, length of follow-up and evaluation methods used to measure outcomes.

Engaging postpartum women, who have time and financial commitments due to young families, in lifestyle interventions is challenging [11–14]. Electronic health (eHealth) technologies are a potential solution for addressing the barriers of time and convenience providing a flexible method of delivery that can be adapted to demands associated with family commitments, thus providing a strategy suitable for scale-up and broad reach [15]. A previous systematic review with meta-analysis (*n*=4 experimental studies, *n*=525 women) identified that eHealth interventions result in a significant reduction in weight among women after 3 to 12 months postpartum, compared to control groups [16]. Recent pilot studies indicate that technology-delivered interventions in postpartum women with previous GDM are also associated with improvements in dietary intake [17], physical activity [17], GDM knowledge [18] and weight status [17, 19].

We previously conducted a pilot RCT to evaluate the feasibility and preliminary efficacy of a 6-month eHealth lifestyle program ‘Body Balance Beyond’, comprising a healthy lifestyle website plus individualised video coaching and text message support [20]. Results indicated that recruiting postpartum women was challenging when the women were required to attend in-person at the research facility to undertake physical and biochemistry measures (e.g., body composition, HbA1C and lipids) at 3 time points. To address this barrier, the current study used self-reported physical measurements collected via survey to reduce participant burden while extending reach by enabling postpartum women from any geographical area to participate. The primary aims of the current study were to: 1) evaluate preliminary intervention effectiveness on weight, diet quality, moderate-vigorous physical activity; 2) assess recruitment and retention of women with recent GDM (feasibility); and 3) evaluate intervention acceptability including satisfaction, usability, appropriateness and usage.

## Methods

### Study design

Body Balance Beyond was a three-arm pilot randomised trial over 3 months in women with recent GDM. The trial was prospectively registered with the Australian New Zealand Clinical Trials Registry (ANZCTR): ACTRN12619000162112. Study design, conduct and reporting were in accordance with the Consolidated Standards of Reporting Trials (CONSORT) guidelines [21], (CONSORT checklist Additional file 1, TIDieR checklist Additional file 2). Process evaluation was embedded within the study to assess intervention feasibility and acceptability. Ethics approval was granted by the University of Newcastle Human Research Ethics Committee (approval number H-2017-0187). Prior to study enrolment, all participants provided electronic informed consent for participation.

### Participants

Participants were recruited March to May 2019. Eligible participants were Australian women, aged 18-45 years, with overweight or obesity (body mass index [BMI] 25-50 kg/m^2^), were greater than 3 months postpartum, and who had been diagnosed with GDM in the past 5 years. This time-period was chosen as the risk of developing T2DM is greatest in the time-period of 3-6 years following a pregnancy affected by GDM [3]. Women were excluded if they were currently pregnant or trying to conceive; had type 1 diabetes mellitus (T1DM) or T2DM, had no medical clearance to exercise or a medical condition or injury that could be exacerbated by exercise, or if they lacked a suitable internet connection (download/upload speed <0.3 Mbps) for conducting video calls [22]. Participants were recruited Australia-wide using the following strategies: 1) media releases and social media posts, and 2) invitation letters emailed to women with previous GDM who were registered on the Diabetes Australia National Diabetes Supply Scheme Register. Interested participants who contacted the research team via email or phone were directed to an online screening questionnaire to assess their eligibility. BMI was assessed using participants’ self-reported current (postpartum) height and weight using data reported during the eligibility screening process. Potential participants who reported having health condition/s that impacted on their ability to exercise were required to seek clearance from their medical practitioner. The online screening questionnaire also included an information statement summarising study requirements, including potential benefits and risks, and a consent form. Recruitment accrual rate was defined as the proportion of women that were randomised in the study compared to those that expressed an interest in the study and were assessed for eligibility. As this was a pilot study, no sample size was calculated, however we aimed to recruit ~30 participants per group. For the current study the program was adapted to be delivered online to evaluate this mode of intervention delivery on retention and completion of the participation requirement.

### Study groups

Eligible participants were randomised (block list randomisation, block size 6; Sealed Envelope Ltd, https://www.sealedenvelope.com) to 1 of 3 groups: High Personalisation (HP), Medium Personalisation (MP) and Low Personalisation (LP).

#### High Personalisation (HP) group

Participants assigned to the HP group received access to a 3-month lifestyle behaviour intervention ‘Body Balance Beyond’ delivered online via the program website which was tailored to women with a history of GDM. Participants received an email with a web-link to the program website and a username and password to log-in. Website resources were predominately self-administered and provided participants with information and tools for supporting healthy lifestyle behaviours related to eating and exercise, stress management and sleep habits. The program website provided 5 content sections which included:

1. *Managing my risk:* identified the risk factors associated with diabetes and strategies for reducing disease risk
2. *My Plan:* participants were emailed personalised feedback reports on their lifestyle behaviours which were used to set goals related to weight, diet and exercise, as well as identifying strategies for self-monitoring and managing relapses.
3. *Eating:* provided healthy eating information and resources including portion size guidelines, energy density, meal planning, reading food labels, recipes, and eating behaviours while breastfeeding.
4. *Physical Activity:* provided information and resources for active living including the health benefits of physical activity, types of physical activity and considerations for being more active.
5. *Wellbeing:* information and resources related to social support, stress management and sleep habits.

In addition to being given access to the program website, participants in the HP group received 5 individual video coaching sessions delivered via video call (20-30 min duration each) with a dietitian (Weeks 2 and 5) and exercise physiologist (Weeks 3 and 6) over the 3 months, with participants selecting the practitioner they wanted to see for their final session (Week 9). Video coaching sessions were delivered via the Zoom© platform (Gartner Inc, California, US, https://zoom.us/). Sessions were based on a structured participant-centered approach implemented in previous studies [20, 23]. Briefly, the respective practitioners reviewed participants’ personalized feedback reports for dietary intake and physical activity levels and developed strategies to overcome self-identified barriers to healthy eating and physical activity reported in the Personalised Nutrition and Personalised Exercise Questionnaires. Following a similar process to a previous study [23], the generic Capacity, Opportunity and Motivation-behaviour model (COM-B) questionnaire [24] was modified to incorporate known barriers associated with healthy eating and physical activity impacting women with young children. In subsequent video coaching sessions the dietitians and exercise physiologists reviewed women’s progress relative to goals and strategies for healthy eating and physical activity and with the participant they problem-solved associated barriers, and revised goals and strategies. Participant attendance to the video coaching sessions was recorded.

Participants assigned to the HP group also received 3 text messages per week sent to their mobile phones. One automated text message prompted participants to self-monitor or reflect on goals while the remaining messages were personalised to address participant barriers to healthy eating and physical activity as identified in their responses to the Personalised Nutrition and Personalised Exercise Questionnaires. For example, a personalised text message which aimed to address meal planning skills as the barrier for healthy eating was: "[First Name]: Looking to improve your menu planning skills? Click here [Link] for tips on creating a healthy menu, and other great advice."

#### Medium Personalisation (MP) group

Participants assigned to the MP group received access to the ‘Body Balance Beyond’ program website and personalised text messages over the 3-month period as described above. No video coaching sessions were provided.

#### Low Personalisation (LP) group

Participants assigned to the LP group received access to ‘Body Balance Beyond’ program website only.

### Outcomes

Self-reported outcome measures were collected at baseline and 3 months via online surveys using Qualtrics (Qualtrics, Seattle, Washington, US), online survey software. All participants were blinded to group allocation until after their baseline assessment. Participants who initially failed to complete their assessment survey were contacted by email and/or phone to remind them to complete their surveys.

#### Weight and height

Weight and height were self-reported by participants at baseline and 3 months. BMI was calculated as weight (kg) / height (m^2^).

#### Diet quality

Dietary intake over the previous 3 months was assessed using the Australian Eating Survey (AES) Food Frequency Questionnaire (FFQ) [25]. The AES has been analysed for reproducibility and comparative validity in Australian adults using 4 day weighed food records for nutrient intake [25], plasma carotenoids for fruit and vegetable intake [26] and red blood cell membrane fatty acids for fatty acid intake [27]. The AES is a self-administered 120-item semi-quantitative questionnaire with an additional 15 demographic and behavioural questions. Portion sizes are calculated for individual food items using data from the Australia Bureau of Statistics National Nutrition Survey [28] or the ‘natural’ serving size of specific foods where appropriate (e.g. a slice of bread). Frequency of consumption was reported by participants using a Likert scale ranging from ‘never’ up to ‘≥7 times per day’, depending on the food. The Australian Nutrient tables (AUSNUT) 2011-13 food composition database was used to compute nutrient intakes [29]. The contribution of specific foods to total energy intake was categorised for nutrient-dense, core food groups (e.g. fruits, vegetables, grains) and energy-dense, nutrient-poor (EDNP), non-core foods (e.g. sweetened drinks, confectionary, takeaway foods) and sub-groups within each of these.

Diet quality was measured using a brief diet quality index, the Australian Recommended Food Score (ARFS) which has demonstrated acceptable reliability and accuracy compared to FFQs and plasma carotenoid concentrations in adults [26, 30]. The ARFS uses a sub-set of 70 questions from the AES that correspond to nutrient-dense core foods recommended in the Australian Dietary Guidelines [31]. The ARFS score is calculated by summing the points from eight subscales, with 20 questions related to vegetable intake, 12 related to fruit, 13 to protein-rich foods (7 to meat and 6 to vegetarian protein sources), 12 to breads/cereals, 10 to dairy and calcium-rich foods, 1 to water, and 2 to spreads/sauces. The total score ranges from zero to a maximum of 73 points with further details published elsewhere [30].

#### Physical activity level

Physical activity levels were assessed using the Godin Leisure-time Exercise Questionnaire (modified version) [32, 33]. Participants reported their usual frequency (times per week) and duration (minimum 10 minutes) of light (minimal physical effort), moderate (not physically exhausting) and vigorous (heart beats rapidly) intensity physical activity they had participated in over the past month. The total time (minutes) of moderate and vigorous physical activity were each multiplied by the frequency and duration and were summed to provide a measure of moderate to vigorous physical activity (minutes/week).

#### Self-efficacy and quality of life

Eating self-efficacy was assessed using the Weight Efficacy Lifestyle Questionnaire - Short Form (WEL-SF), an 8-item scale which evaluates participants’ level of confidence to resist eating in a variety of situations and emotional states [34]. Each scale item was scored on an 11-point Likert scale from zero (not confident at all) to 10 (very confident). Scores from each scale item were summed to provide the total score ranging from 0-80. The WEL-SF has demonstrated acceptable reliability and construct validity in a sample of patients (18-80 years) with obesity [34, 35].

Exercise self-efficacy was assessed using the Self-Efficacy for Exercise scale (SEE), a 9-item scale which evaluates participants’ level of confidence to exercise 3 times a week for 20 minutes under nine different situations [36]. For each situation the participant was scored on an 11-point scale from zero (not confident at all) to ten (very confident). Scores from each scale item were summed to provide the total score ranging from zero to 90. The SEE has demonstrated acceptable reliability (Cronbach’s alpha = 0.92) and construct validity in a sample of adults (*n*=187) [36].

Quality of life was assessed using the Assessment of Quality of Life 6-dimension instrument (AQoL-6D) [37], a 20-item scale assessing 6 domains including independent living, relationships, mental health, coping, pain and senses, as well as a global ‘utility’ score. The AQoL-6D has demonstrated acceptable construct validity, criterion validity and test-retest reliability in a sample of adults aged 18-44 years [38, 39].

#### Process evaluation

All participants completed an online process evaluation survey via Qualtrics (Qualtrics, Seattle, Washington, US) at 3 months to assess program acceptability, including satisfaction, usability, appropriateness and program usage. Participants reported on their experiences and engagement with relevant intervention components (HP: ‘Body Balance Beyond’ website and goal setting module, AES and physical activity reports, video coaching sessions, text messages; MP: ‘Body Balance Beyond’ website and goal setting module, AES and physical activity reports, text messages; LP: ‘Body Balance Beyond’ website and goal setting module, AES and physical activity reports). The survey questions assessed the following aspects using a 5-point Likert scale (unless specified) using methods described previously [40, 41] :

##### Satisfaction

Participants reported their level of satisfaction with each intervention component.

##### Usability

Participants rated the usability (e.g., ‘was easy to navigate’) and ability to engage (e.g., ‘made me feel accountable’) in all intervention components and were also asked to rate the appeal of the program website (e.g., ‘was visually appealing) and their experience of the video coaching sessions (e.g., ‘the picture quality of the video coaching sessions was acceptable’). Participants were asked whether they experienced any issues assessing and using the video coaching sessions (yes/no) and whether the intensity (number and duration) of the video coaching sessions was appropriate (just right/ preferred more/ preferred less).

##### Appropriateness

Participants rated the relevance of the content presented in the ‘Body Balance Beyond’ website, the video coaching sessions and the text messages (e.g., ‘provided me with useful information about healthy eating’). Participants were also asked to rate the scheduling process for the video coaching sessions (‘the scheduling of the video coaching sessions was appropriate’).

##### Usage

Participants were asked whether they read/saw the AES and Physical Activity reports, accessed the ‘Body Balance Beyond’ website or attended video coaching sessions (HP group only). Participants were also asked about what they liked and did not like about each component of the program and were given the opportunity to provide additional feedback on intervention components and the program overall.

#### Demographic characteristics, pregnancy/GDM history and health conditions

Sociodemographic information and data relating to participants’ pregnancy, GDM history and health conditions were collected via questionnaire at baseline, including age, date of birth, country of birth, highest educational qualification completed, Aboriginal/Torres Strait Islander status, marital status, household income, self-reported ability to manage on current household income (scored on a 5-point scale from ‘Impossible’ to ‘Easy’) and current smoking status.

### Statistical analysis

Statistical analyses were conducted using SPSS for Windows version 25.0 (SPSS Inc, Chicago, Illinois, US). Demographic and baseline characteristics were reported for participants across the 3 study groups as mean (standard deviation, SD) for continuous variables and percentages (counts) for categorical variables.

Generalised linear mixed models were used to analyse the outcomes for the impact of treatment (HP vs. MP vs. LP , time (baseline, 3 months) and the treatment-by-time interaction. Treatment group, time and the treatment-by-time interaction formed the 3 terms for the base model, ensuring that participants who withdrew prior to 3 months were retained in analyses as consistent with an ‘intention-to-treat’ approach. Base models were initially tested using compound symmetry and unstructured variance types, and the appropriate variance type was selected for each model using the Akaike Information Criterion. Age and BMI were also included as covariates to determine any significant interactions in the models for each outcome. Where a covariate was significant, two-way interactions with time and treatment were examined, and any significant two-way interactions were adjusted for in the model. When a three-way interaction with the covariate by group-by-time was significant, this three-way interaction and all relevant two-way interactions were adjusted for in the model [42]. Coefficients and p-values for the group-by-time interaction term were assessed to determine the efficacy of the intervention. A significance level of *p*<0.05 was set for the primary and all secondary outcomes.

For the process evaluation survey, frequency data for each response option and qualitative data from the open-ended questions were reported at 3 months for the LP, MP and HP groups.

## Results

### Recruitment and retention (Feasibility)

**Fig. 1:**
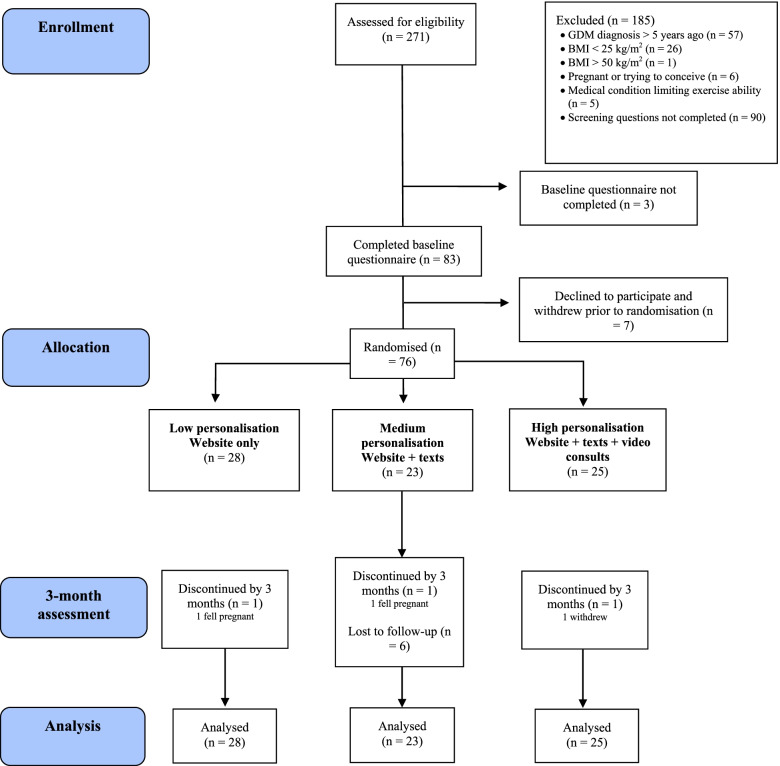
CONSORT diagram describing study design and flow of participants through the 3-month randomised trial of the *Body Balance Beyond* eHealth intervention for women with recent gestational diabetes mellitus

Participant flow though the trial is reported in Fig. 1. From the screening survey, 188 of 271 women were deemed eligible for the study, of whom 83 completed the baseline questionnaire. Seventy-six women were randomised to HP (*n*=25), MP (*n*=23) and LP (*n*=28), achieving a 28% recruitment accrual rate.

Overall, 53 (70%) women completed the study. Retention at 3 months was 84% (*n*=21) for HP, 70% (*n*=16) for MP and 57% (*n*=16) for LP. Reasons for dropouts included becoming pregnant (*n*=1); withdrew with no reason given (*n*=2); lost to follow-up (*n*=20).

### Participants

Characteristics of the study sample across the 3 groups are reported in Table 1. Mean age was 36.0 ± 5.0 y. The majority of women were born in Australia (75%, *n*=57), two-thirds had a university degree (67%, *n*=51) and most were married (93%, *n*=71). Mean time since first GDM diagnosis was 45.5 ± 28.5 months. Mean weight was 85.2 ± 16.0 kg and BMI was 31.1 ± 4.8 kg/m^2^.

**Table 1:** Baseline characteristics of women randomised to the 3 study groups in a randomised trial of the *Body Balance Beyond* eHealth intervention for women with recent GDM (*n* = 76)

Sociodemographic characteristics	HP Group *n* = 25	MP Group *n* = 23	LP Group *n* = 28
Age (years) mean(SD)	36.0 ± 5.4	34.6 ± 5.3	37.3 ± 4.2
Country of birth			
Australia	18 (72)	19 (83)	20(71)
Highest qualification completed			
School/Higher School Certificate	2 (8)	0 (0)	0 (0)
Certificate/diploma/ trade	7 (28)	6 (26)	10(36)
University degree	16 (64)	17 (74)	18 (64)
Aboriginal/Torres Strait Islander status			
Aboriginal	1 (4)	0 (0)	0 (0)
Torres Strait Islander	0 (0)	0 (0)	0 (0)
Marital status			
Married/De-facto	24 (96)	22 (96)	25 (89)
Separated/divorced	1 (4)	1(4)	1 (4)
Never married	0 (0)	0 (0)	2 (7)
Ability to manage on current income			
Difficult all/Some of the time	11 (44)	14 (61)	12 (43)
Not too bad/Easy	14 (56)	9 (39)	16 (57)
Smoking status			
Non-smoker	25 (100)	21 (91)	27 (96)
Current smoker	0 (0)	2 (9)	1 (4)
**Pregnancy and GDM history**			
Parity Mean (SD)	2.2 ± 1.0	2.2 ± 1.1	1.9 ± 0.8
Months since first GDM diagnosis Mean (SD)	56.8 ± 38.4	40.6 ± 19.7	39.3 ± 21.1
GDM in subsequent pregnancies			
Yes	10 (40)	4 (17)	4 (14)
No	5 (20)	4 (17)	4 (14)
Not applicable (no subsequent pregnancies since being diagnosed)	10 (40)	15 (65)	20 (71)
**GDM management**			
Diet	25 (100)	19 (83)	24 (86)
Exercise	13 (52)	12 (52)	13 (46)
Tablets	5 (20)	2 (9)	5 (18)
Insulin	9 (36)	12(52)	11 (39)
**Other health conditions**			
High blood pressure	2 (8)	4 (17)	6 (21)
High cholesterol	1 (4)	2 (9)	1 (4)
Preeclampsia	1(4)	5 (22)	4 (14)
PCOS	5 (20)	5 (22)	2 (7)
Impaired glucose tolerance or impaired fasting glucose	2 (8)	2 (9)	4 (14)
High free thyroid hormone levels (hyperthyroidism)	1 (4)	1(4)	2 (7)
Low free thyroid hormone levels (hypothyroidism)	4 (16)	2 (9)	3 (11)
**Anthropometry** mean(SD)			
Height (m)	1.6 ± 0.1	1.7 ± 0.1	1.7 ± 0.1
Weight (kg)	82.3 ± 12.3	89.8 ± 14.6	84.0 ± 19.4
BMI (kg/m^2^)	30.7 ± 4.3	32.8 ± 5.1	30.0 ± 4.8
BMI classification			
Overweight (≥ 25 to ≤ 30kg/m^2^)	14 (56)	8 (35)	16(57)
Obese (>30 kg/m^2^)	11 (44)	15 (65)	12 (43)
**Dietary intake** mean (SD)			
ARFS total score	34.1 ± 9.2	32.4 ± 11.8	35.3 ± 9.5
ARFS vegetables	12.4 ± 5.0	12.7 ± 5.4	13.9 ± 4.5
ARFS fruits	5.8 ± 3.0	4.1 ± 8.4	5.6 ± 3.1
ARFS meat	3.0 ± 1.6	2.3 ± 1.0	2.1 ± 1.2
ARFS protein alternatives^a^	2.2 ± 1.5	2.6 ± 2.1	2.1 ± 1.4
ARFS grains	5.6 ± 2.0	5.5 ± 2.6	5.8 ± 1.9
ARFS dairy	3.3 ± 1.7	3.1 ± 1.9	4.1 ± 1.8
ARFS water	0.6 ± 0.5	0.9 ± 0.3	0.7 ± 0.5
ARFS extras^b^	1.2 ± 0.9	1.1 ± 0.8	1.0 ± 0.7
% energy – five core food groups^c^	63.6 ± 11.2	57.6 ± 12.2	58.9 ± 13.4
% energy – non-core foods^d^	36.4 ±11.3	42.4 ± 12.2	41.4 ± 13.4
**Physical activity** mean (SD)			
MVPA (minutes/week)	95.5 ± 140.4	91.4 ± 91.2	80.5 ± 84.6
**Weight self-efficacy** mean (SD)			
WEL-SF total score	40.9 ± 15.0	38.4 ± 15.7	40.8 ± 15.9
**Exercise self-efficacy** mean (SD)			
Ex-SE total score	42.0 ± 14.7	44.2 ± 14.5	40.4 ± 19.8
**Eating behaviours** mean (SD)			
TFEQ-R18 Cognitive restraint score	18.0 ± 1.8	16.6 ± 1.8	18.0 ± 2.6
TFEQ-R18 Uncontrolled eating	23.2 ± 3.9	24.8 ± 4.2	23.7 ± 3.2
TFEQ-R18 Emotional eating	7.0 ± 2.5	7.5 ± 2.0	7.4 ± 2.4
**Quality of life** mean (SD)			
AQoL-6D utility (global) score	0.83 ± 0.13	0.73 ± 0.18	0.75 ± 0.13
AQoL-6D independent living score	0.93 ± 0.15	0.93 ± 0.15	0.98 ± 0.031
AQoL-6D relationships score	0.80 ± 0.24	0.80 ± 0.24	0.83 ± 0.18
AQoL-6D mental health score	0.48 ± 0.28	0.48 ± 0.28	0.53 ± 0.24
AQoL-6D coping score	0.65 ± 0.28	0.65 ± 0.28	0.53 ± 0.22
AQoL-6D pain score	0.77 ± 0.26	0.77 ± 0.26	0.75 ± 0.24
AQoL-6D senses score	0.96 ± 0.07	0.96 ± 0.07	0.95 ± 0.10

Data is presented as n (%) or mean (SD)

Abbreviations: *AQoL-6D* Assessment of Quality of Life instrument 6-dimension, *ARFS* Australian Recommended Food Score, *BMI* Body mass index, *Ex-SE* Exercise Self-Efficacy Scale, *GDM* Gestational diabetes mellitus, *MVPA* Moderate to vigorous physical activity, *mYFAS* Modified Yale Food Addiction Score, *PCOS* Polycystic ovarian syndrome, *SD* Standard deviation, *TFEQ-R18* Three-Factor Eating Questionnaire-R18, *WEL-SF* Weight Efficacy Lifestyle Questionnaire

^a^ARFS protein alternatives: includes vegetarian protein-rich food sources (e.g. eggs, tofu, lentils, legumes, nuts). ^b^ARFS extras: energy-dense nutrient-poor foods that are not classified in the five core food groups

^c^% energy – core foods: percentage of energy derived from the five core-food groups (e.g. grains, vegetables and legumes, fruit, dairy and alternatives and meat and alternatives)

^d^% energy – non-core foods: percentage of energy derived from energy-dense nutrient poor foods that are not classified in the five core food groups

### Preliminary efficacy

Table 2 summarises the results of the intention-to-treat analyses for within- and between-group changes from baseline to 3 months on preliminary efficacy outcomes. From baseline to follow-up, mean WEL-SF scores in the HP group increased (improved) by 9.71 (95% confidence interval (95%CI): 9.27, 10.15) points (on a scale from 0-80). The mean increase in WEL-SF scores in the HP group was significantly greater compared to the MP and LP groups (mean [95% CI] difference between groups 13.29 [2.77, 23.82] points and 11.06 [0.63, 21.49] points, respectively; *P*=.03). From baseline to follow-up, mean AQoL-6D Utility (global) scores in the HP group increased (improved) by 0.07 (95% CI: 0.07, 0.08) points (on a scale from 0-1). The mean increase in AQoL-6D utility scores in the HP group was significantly higher compared to the MP and LP groups (mean [95% CI] difference between groups 0.08 [0.01, 0.15] points and 0.09 [0.01, 0.16] points, respectively; *P*=.03). There were no significant group-by-time effects observed for any of the remaining outcomes (weight, ARFS, % energy from core foods, moderate to vigorous physical activity, exercise self-efficacy) (all *P*>.05). Although percentage of energy intake from nutrient-dense core foods improved for all intervention groups, there was no significant difference among the groups (HP:36.4%; MP:42.4%; LP:41.4%, *P*>.05) (Table 2)). Exercise self-efficacy was the only outcome for which a two-way interaction was significant (age x time), with no change to the results when this two-way interaction was included in the model (see Supplementary file 2).

**Table 2:** Mean (95% CI) change in outcomes within groups and among groups (intention-to-treat population) from baseline to 3-months in a randomised trial of an eHealth lifestyle intervention for women with recent GDM.

Outcome	HP Group *n* = 25	MP Group *n* = 23	LP Group *n* = 28	MP vs LP groups	HP vs LP groups	HP vs MP groups	***P***-value
Weight (kg)	-1.99 (-2.00, -1.97)	-1.93 (-1.97, -1.89)	-1.27 (-1.33, -1.21)	-0.66 (-2.47, 1.14)	-0.72 (-2.41, 0.98)	-0.06 (-1.76, 1.64)	.660
ARFS (max. 73)^c^	-0.89 (-1.37, -0.42)	1.09 (0.53, 1.64)	-0.31 (-1.4, 0.8)	0.41 (-5.06, 5.87)	1.92 (-3.24, 7.08)	-1.92 (-7.08, 3.24)	.73
% energy- core foods^d^	6.76 (5.95, 7.57)	6.06 (5.13, 7.00)	7.36 (5.48, 9.24)	0.68 (-7.35, 8.72)	-0.46 (-8.09, 7.16)	0.46 (-7.16, 8.09)	.95
MVPA (minutes/week)	26.77 (23.52, 30.02)	-23.97 (-31.41, -16.52)	64.70 (52.18, 77.21)	-88.66 (-167.73, -9.59)	-37.93 (-113.02, 37.17)	50.73 (-24.34, 125.81)	.08
WEL-SF (max. 80)	9.71 (9.27, 10.15)	-3.59 (-4.60, -2.58)	-1.35 (-2.86, 0.15)	-2.23 (-13.27, 8.80)	**11.06** (0.63, 21.49)*	**13.29** (2.77, 23.82)*	**.03**^**e**^
Ex-SE^c^ (max. 90)	5.51(5.03, 5.99)	-4.81(-5.86, -3.76)	-4.11 (-5.71, -2.50)	-0.70(-1.26, -0.15)	9.62(8.49, 10.75)	10.32(9.75, 10.90)	.12
AQoL-6D Utility^d^ (max 1)	0.07 (0.07, 0.08)	-0.01 (-0.01, 0.00)	-0.01 (-0.02, 0.00)	0.01 (-0.07, 0.09)	**0.09** (0.01, 0.16)*	**0.08** (0.01, 0.15)*	**.03**^**e**^

Abbreviations: *AQoL* Assessment of Quality of Life 6-dimension, *ARFS* Australian Recommended Food Score, *BMI* Body mass index, *CI* Confidence interval, *Ex-SE* Exercise Self-Efficacy Scale, *GDM* Gestational diabetes mellitus, *HP* High Personalisation Group, *LP* Low Personalisation Group, *Max* Maximum score, *MP* Medium Personalisation Group *MVPA* Moderate to vigorous physical activity, *WEL-SF* Weight Efficacy Lifestyle Questionnaire- short form

^a^ Time differences were calculated as 3 months minus baseline

^b^ Between-group differences in changes from baseline to 3 months

^c^ Adjusted for age

^d^ Adjusted for BMI

^e.^ *P < .05*

Among those who completed the study, 38 women (72% of completers: HP *n*=17 [81%], MP *n*=12 [75%], LP *n*=9 [56%]) lost weight at 3 months. Among those who lost weight, mean weight loss was 2.8 ± 2.0 kg (HP: 2.6 ± 1.6 kg; MP: 2.8 ± 1.7 kg; LP: 3.3 ± 3.1 kg). Ten women (19% of completers: HP *n*=4; MP *n*=3; LP *n*=3) lost ≥5% of their body weight at 3 months. There was no difference among groups in the proportion of participants who lost weight or who lost ≥5% of their body weight (*P*>.05).

### Acceptability

#### ‘Body Balance Beyond’ program overall (all groups)

Two-thirds of women in the HP group (*n*=14) were satisfied with the program overall and 86% (*n*=18) would recommend it to others, compared with 25% (*n*=4) and 44% (*n*=7) in the MP group, and 14% (*n*=2) and 36% (*n*=5) in the LP group, respectively.

## Discussion

This randomised trial evaluated the preliminary efficacy, feasibility and acceptability of a 3-month eHealth lifestyle program, Body Balance Beyond, both with and without video coaching sessions and/or text messages among women with recent GDM. Improvements were observed in self-efficacy and quality of life scores which favoured the HP group, although not in any other outcomes. Feasibility was challenging as indicated by a 28% recruitment accrual rate and a 70% retention rate, although retention was highest in the HP group (84%). Based on findings from the process evaluation surveys, acceptability (satisfaction, usability, appropriateness and usage) of the program appeared to be much higher in the HP group compared to the MP and LP groups.

The sample of women who were enrolled in the trial (*n*=76) was higher compared to our previous pilot RCT (*n*=42) but a recruitment accrual rate of only 28% was achieved [20]. Nicklas et al. [43] conducted a similar lifestyle intervention trial in women with recent GDM and achieved a recruitment accrual rate of 69%. This outcome was attributed to the use of an in-person recruiter, integrating recruitment in a clinical setting, developing relationships with patients, allowing for flexibility in recruitment and minimising barriers to participation [43]. In contrast, the current study used less costly passive methods, including media releases, social media posts and email invitation from the Diabetes Australia National Diabetes Supply Scheme Register, which may have contributed to a less effective recruitment strategy. Previous studies that have attempted to recruit women during the postpartum period have reported similar findings [44–46]. Furthermore, a recent Cochrane review of 29 trials identified that how trial information is communicated such as the format (i.e. written versus verbal), delivery and timing; and perceptions of trial elements including randomisation and withdrawal process were factors that significantly influenced study participation [47]. This suggests that a combination of strategies may be needed to ensure that recruitment is person-centred but also adaptable based on the needs of postpartum women.

The current study achieved a retention rate of 70% at 3 months which lower than expected compared to our previous pilot study which achieved a retention rate of 71% at 6 months [20] and previous 3-12 month lifestyle intervention studies among women with recent GDM (69-91%) [11–14]. Although one of these studies did provide monetary incentives to the participants to defray costs of transportation, parking and childcare [13]. Dropout was higher in the LP and MP groups compared to the HP group which suggests that interest in the study intervention was higher in women who received one-to-one health professional support from video coaching sessions. This is supported by the findings of Mahen et al. [48] in which a 20% improvement in the attrition rate post-treatment was achieved by providing weekly telephone support from mental health workers to help guide women through a 12 module web-based program for postpartum depression. Interestingly, Lim et al. [49] reported that women with a history of GDM who expressed higher levels of engagement in a lifestyle intervention had greater resilience and resourcefulness to overcome barriers associated with participating in the program compared to women who were not engaged [49]. Overall, these findings suggest that interventions that aim to provide sufficient support and improve resilience and resourcefulness may increase participation and engagement in this population.

Results from the current trial are promising regarding the impact of the eHealth lifestyle program on the health outcomes of women with a history of GDM. Significant improvements in quality of life were found in the HP group compared to the MP and LP groups. These findings are supported by a Cochrane review [50] in which personalised care was associated with improvements in physical and psychological health status compared to usual care in adults managing chronic diseases. These improvements are important considering that the diagnosis of GDM is associated with a decline in quality of life in women during pregnancy [51]. Furthermore, women in the HP group achieved a significant improvement in mean WEL-SF scores, a measure of confidence to resist eating in a variety of situations as well as emotional states, which may have impacted positively on their quality of life scores. This is consistent with the findings of Mitchison et al. [52] in which binge eating in Australian men and women (*n*=3010 in 1998 *n*=3034 in 2008) predicted a significant decline in quality of life score at 2 time-points (*P*=.001).

In the women who lost weight (72%, mostly in the HP and MP groups), the mean weight loss was 2.8kg. Similarly, in our previous pilot study we observed that the women who lost weight on average reduced their body weight by 3.1 kg at 6 months [20]. This is clinically important as women with a history of GDM tend to retain pregnancy related weight or gain weight during the postpartum period [53]. A meta-analysis of nine lifestyle interventions studies, including 2 web-based interventions for women with a history of GDM, was associated with weight loss (mean difference (MD):−1.07 kg; 95% confidence interval (CI 95%) −1.43 to −0.72 kg) [8]. Although the effect on weight was small in the current study, one study reported that weight loss of 1.6 kg in women with a history of GDM (*n*=350) was associated with a 53% risk reduction in T2D 3 years after diagnosis [54]. We acknowledge that the current sample included women who were between 3 months and 5 years postpartum therefore, the outcome of weight loss may have been affected by the time since GDM diagnosis. However, this limitation reflects the pragmatic approach of the study to meet the requirements for potential scalability of the intervention. The consumption of core foods improved in all intervention groups, which may have contributed to the weight loss observed in the sample. This observed improvement in diet quality is significant as Tobias et al. [55] reported that the highest adherence to healthy dietary patterns (i.e. alternate Mediterranean diet, Dietary Approaches to Stop Hypertension, and alternate Healthy Eating Index) among women with a history of GDM (*n*=4413) were associated with a 50-57% lower risk of T2DM compared with the lowest adherence over 16 years of follow-up. Satisfaction ratings for the Body Balance Beyond program were higher in the HP group compared to the MP and LP groups, although differences were not evaluated statistically due to small group sizes. This could be attributed to the addition of personalised one-on-one health professional support received by the HP group during video coaching sessions as most women found these sessions to be informative and increased their confidence levels for changing health behaviours. However, further research is needed from future RCTs to confirm this finding.

### Strengths and limitations

Strengths of the current study included the randomised design and that intervention development was based on formative research conducted in the target population [20]. This study used web-based delivery for the intervention and removed the need for in-person visits to improve engagement and reduce barriers to participation in postpartum women. However, the study findings should be interpreted with caution as outcome measures were based on self-reported data, therefore the possibility of recall bias and misreporting error cannot be excluded. To reduce participant burden, the study was limited to self-reported measures which meant that the impact of the intervention on biochemical outcome measures (e.g. HbA1c, fasting glucose) could not be determined. This pilot study did not include a control group therefore, the magnitude of the intervention effect is more difficult to interpret. Due to the short duration of the intervention (3 months) it is unclear whether any improvement would be maintained over time. The sample recruited may not represent women of a wide range of risk for developing T2DM as women that had already developed T2DM were excluded from the study. Furthermore, in the sample the mean time since first GDM diagnosis was 45.5 months, which may indicate that women were at higher risk of developing T2DM due to the length in time since they had a pregnancy affected by GDM. Since T2DM risk was not quantified for each participant it is difficult to ascertain the range of risk represented by those recruited into the current study. A high proportion (76%) of the sample had attained a university degree, which is significantly higher than the 2019 national data where 31% of females attained a university degree or higher [56]. Although O’Reilly et al. [12] also reported a high proportion (84%) of women as having attained tertiary education in a sample of Australian women (*n*=573) and a history of GDM within their first postnatal year who were participating in an RCT focusing on postnatal diabetes prevention. This study also analysed website usage using self-reported data and therefore these findings should be interpreted with caution.

## Conclusion

The results from this pilot randomised trial demonstrate feasibility and elements of efficacy on quality of life and wellbeing of the eHealth ‘Body Balance Beyond’ program in postpartum women with a history of GDM. Findings from the process evaluation indicate that the program was highly acceptable in the HP group, however further refinement of the website and text messages is needed to improve engagement and efficacy.

## Supplementary Information

**Additional file 1.** CONSORT checklist.

**Additional file 2.** TIDieR checklist.

**Additional file 3.** Process evaluation.

**Additional file 4: Supplementary file 2.** Results for Exercise Self-Efficacy Scale model including two-way interaction of age x time.

## Data Availability

The datasets generated and/or analysed during the current study are not publicly available as this was not stated to participants who completed the study

## Abbreviations

AES: Australian Eating Survey
AQoL-6D: Assessment of Quality of Life 6-dimension instrument
ANZCTR: Australian New Zealand Clinical Trials Registry
AUSNUT: Australian Nutrient tables
ARFS: Australian Recommended Food Score
BMI: Body mass index
COM-B: Capability, Opportunity and Motivation-behaviour model
CONSORT: Consolidated Standards of Reporting Trials
EDNP: Energy-dense, nutrient-poor
eHealth: Electronic health
FFQ: Food Frequency Questionnaire
GDM: Gestational diabetes mellitus
HbA1c: Haemoglobin A1c
HDL: High-density lipoprotein
HP: High Personalisation
JMIR: Journal of Medical Internet Research
LDL: low-density lipoprotein
LP: Low Personalisation
MD: Mean difference
MP: Medium Personalisation
RCT: Randomised controlled trial
T1DM: Type 1 diabetes mellitus
T2DM: Type 2 diabetes mellitus
WEL-SF: Weight Efficacy Lifestyle Questionnaire-Short Form
