# Supplementary material for: Evaluation of a Type 2 diabetes risk reduction online program for women with recent gestational diabetes: a randomised trial
Source: Int J Behav Nutr Phys Act. 2022 Mar 28;19:35. doi: 10.1186/s12966-022-01275-3 (PMC8962548; doi:10.1186/s12966-022-01275-3)
Supplement: Supplementary file 3 — Additional file 3. Process evaluation. [file 12966_2022_1275_MOESM3_ESM.docx]

**Supplementary table 1.**Frequency of responses to process evaluation questions for the High Personalisation, Medium Personalisation and Low Personalisation intervention groups in a 3-month pilot randomised trial for women with recent GDM who were at risk of developing T2D (*n*=53).

| **Program element** | **Question** | **HP**  **Group**  **n = 21** | **MP**  **Group**  **n = 16** | **LP**  **Group**  **n = 14^a^** |
| --- | --- | --- | --- | --- |
|  |  | n (%) | n (%) | n (%) |
| **Australian Eating Survey report** | I read/saw the Australian Eating Survey report |  |  |  |
|  | Yes | 19 (91) | 11 (69) | 11 (79) |
|  | No | 2 (9) | 5 (31) | 3 (21) |
|  | Easy to understand^b^ |  |  |  |
|  | Agree/Strongly agree  Neutral  Disagree/Strongly disagree | 14 (74)  4 (21)  1 (5) | 10 (91)  1 (9)  0 | 8 (73) |
|  |  |  |  | 2 (18) |
|  |  |  |  | 1 (9) |
|  | Helped identify areas in my diet to improve^b^ |  |  |  |
|  | Agree/Strongly agree  Neutral  Disagree/Strongly disagree | 17 (90)  1 (5)  1 (5) | 11 (100)  0  0 | 7 (64)  3 (27)  1 (9) |
|  | Helped identify areas in my diet where I am doing well^b^ |  |  |  |
|  | Agree/Strongly agree  Neutral  Disagree/Strongly disagree | 17 (90)  1 (5)  1 (5) | 10 (91)  1 (9)  0 | 7 (64)  3 (27)  1 (9) |
|  | Helped me set diet goals^b^ |  |  |  |
|  | Agree/Strongly agree  Neutral  Disagree/Strongly disagree | 16 (84)  2 (11) | 9 (82)  1 (9)  1 (9) | 5 (46)  4 (36)  2 (18) |
| **Physical Activity report** | I read/saw the Physical Activity Report |  |  |  |
|  | Yes  No | 16 (76) | 8 (50) | 5 (36) |
|  |  | 5 (24) | 8 (50) | 9 (64) |
|  | Easy to understand^b^ |  |  |  |
|  | Agree/Strongly agree  Neutral  Disagree/Strongly disagree | 13 (81)  3 (19)  0 | 6 (75)  1 (13)  1 (12) | 8 (89)  0  1 (11) |
|  | Helped me identify areas in my physical activity to improve^b^ |  |  |  |
|  | Agree/Strongly agree  Neutral  Disagree/Strongly disagree | 15 (94) | 6 (75) | 8 (89) |
|  |  | 1 (6)  0 | 1 (13)  1 (12) | 0  1 (11) |
|  | Helped me identify areas in my physical activity where I am doing well^b^ |  |  |  |
|  | Agree/Strongly agree  Neutral  Disagree/Strongly disagree | 16 (100)  0  0 | 4 (50)  3 (38)  1 (12) | 6 (67)  2 (22)  1 (11) |
|  | Helped me set physical activity goals^b^ |  |  |  |
|  | Agree/Strongly agree  Neutral  Disagree/Strongly disagree | 16 (100)  0  0 | 5 (63)  1 (13)  1 (12) | 6 (67)  0  3 (33) |
| **Body Balance Beyond website: Content** | I accessed the BBB website |  |  |  |
|  | Yes | 17 (81) | 13 (81) | 10 (71) |
|  | No | 4 (19) | 3 (19) | 4 (29) |
|  | Provided me with useful information about my diabetes risk^b^ |  |  |  |
|  | Agree/Strongly agree  Neutral  Disagree/Strongly disagree | 14 (82)  3 (18)  0 | 9 (69)  1 (8)  3 (23) | 6 (60)  3 (30)  1 (10) |
|  | Provided me with useful information about healthy eating^b^ |  |  |  |
|  | Agree/Strongly agree  Neutral  Disagree/Strongly disagree | 13 (77)  2 (12)  2 (12) | 7 (54)  3 (23)  3 (23) | 5 (50)  3 (30)  2 (20) |
|  | Provided me with useful information about physical activity^b^ |  |  |  |
|  | Agree/Strongly agree  Neutral  Disagree/Strongly disagree | 13 (77)  3 (18)  1 (6) | 7 (54)  3 (23)  3 (23) | 5 (50)  3 (30)  2 (20) |
|  | Provided me with useful information about weight-loss strategies^b^ |  |  |  |
|  | Agree/Strongly agree  Neutral  Disagree/Strongly disagree | 12 (71)  3 (18)  2 (12) | 5 (39)  5 (39)  3 (23) | 5 (50)  3 (30)  2 (20) |
|  | Provided me with useful information about other wellbeing strategies^b^ |  |  |  |
|  | Agree/Strongly agree  Neutral  Disagree/Strongly disagree | 10 (59)  6 (35)  1 (6) | 6 (46)  4 (31)  3 (23) | 2 (20)  4 (40)  4 (40) |
|  | Helped me to attain my goals^b^ |  |  |  |
|  | Agree/Strongly agree  Neutral  Disagree/Strongly disagree | 8 (47) | 0 | 1 (10) |
|  |  | 4 (24)  5 (29) | 8 (62)  5 (38) | 4 (40)  5 (50) |
|  | BBB website was motivating^b^ |  |  |  |
|  | Agree/Strongly agree  Neutral  Disagree/Strongly disagree | 7 (41)  5 (29)  5 (30) | 1 (8)  6 (46)  6 (46) | 2 (20)  3 (30)  5 (50) |
|  | Made me feel accountable^b^ |  |  |  |
|  | Agree/Strongly agree  Neutral  Disagree | 6 (35)  6 (35)  4 (24) | 3 (23)  3 (23)  7 (54) | 1 (10)  4 (40)  4 (40) |
|  | Easy to navigate^b^ |  |  |  |
|  | Agree/Strongly agree  Neutral  Disagree/Strongly disagree | 12 (71)  3 (18)  2 (12) | 7 (54)  2 (15)  4 (31) | 6 (60)  1 (10)  3 (30) |
|  | Visually appealing^b^ |  |  |  |
|  | Agree/Strongly agree  Neutral  Disagree/Strongly disagree | 13 (77)  2 (12)  2 (12) | 7 (54)  3 (23)  3 (23) | 2 (20)  4 (40)  4 (40) |
|  | Satisfaction with managing my risk^b^ |  |  |  |
|  | Satisfied/Very satisfied  Neutral  Unsatisfied/Very unsatisfied  Did not access this section | 10 (59)  4 (24)  0  3 (18) | 8 (62)  3 (23)  2 (15)  0 | 5 (50)  2 (20)  2 (20)  1 (10) |
|  | Satisfaction with developing my plan^b^ |  |  |  |
|  | Satisfied/Very satisfied | 11 (65) | 5 (39) | 3 (30) |
|  | Neutral  Unsatisfied/Very unsatisfied | 4 (24)  1 (6) | 5 (39)  3 (23) | 3 (30)  2 (20) |
|  | Did not access this section | 1 (6) | 0 | 1 (10) |
|  | Satisfaction with healthy eating resources^b^ |  |  |  |
|  | Satisfied/Very satisfied  Neutral  Unsatisfied/Very unsatisfied  Did not access this section | 11 (65)  4 (24)  1 (6)  1 (6) | 6 (46)  5 (39)  2 (15)  0 | 4 (40)  2 (20)  3 (30)  1 (10) |
|  | Satisfaction with physical activity resources^b^ |  |  |  |
|  | Satisfied/Very satisfied  Neutral  Unsatisfied/Very unsatisfied  Did not access this section | 10 (59)  5 (29)  1 (6)  1 (6) | 7 (54)  5 (39)  1 (8)  0 | 5 (50)  2 (20)  2 (20)  1 (10) |
|  | Satisfaction with wellbeing resources^b^ |  |  |  |
|  | Satisfied/Very satisfied  Neutral  Unsatisfied/Very unsatisfied  Did not access this section | 9 (53)  5 (29)  0  3 (18) | 5 (39)  5 (39)  3 (23)  0 | 2 (20)  5 (50)  2 (20)  1 (10) |
| **Body Balance Beyond website: Goal Setting Module** | Frequency using the Goal Setting Module |  |  |  |
|  | At least once per month | 12 (71) | 7 (54) | 4 (40) |
|  | Never | 5 (29) | 6 (46) | 6 (60) |
|  | Made it easy to set weight-related goals  Agree/Strongly agree  Neutral  Disagree/Strongly disagree |  |  |  |
|  |  | 11 (92)  1 (8)  0 | 4 (67)  0  2 (33) | 2 (50)  2 (50)  0 |
|  | Made it easy to set my nutrition goals |  |  |  |
|  | Agree/Strongly agree  Neutral     Disagree/Strongly disagree | 11 (92)  1 (8)  0 | 5 (83)  1 (17)  0 | 3 (75)  1 (25)  0 |
|  | Made it easy to set my exercise goals |  |  |  |
|  | Agree/Strongly agree  Neutral  Disagree/Strongly disagree | 9 (75)  3 (25)  0 | 5 (83)  1 (17)  0 | 2 (50)  2 (50)  0 |
|  | I liked being able to select from pre-written goals |  |  |  |
|  | Agree/Strongly agree  Neutral  Disagree/Strongly disagree | 11 (92) | 5 (83) | 4 (100) |
|  |  | 1 (8)  0 | 1 (17)  0 | 0  0 |
|  | Email summary of my goals was motivating |  |  |  |
|  | Agree/Strongly agree  Neutral  Disagree/Strongly disagree | 9 (75)  2 (17)  1 (8) | 3 (50)  0  3 (50) | 2 (50)  2 (50)  0 |
|  | Email summary of my goals was useful |  |  |  |
|  | Agree/Strongly agree  Neutral  Disagree/Strongly disagree | 10 (84)  1 (8)  1 (8) | 2 (33)  2 (33)  2 (33) | 3 (75)  1 (25)  0 |
| **Video coaching sessions: dietitian**^c^ | Provided me with useful information about nutrition |  |  |  |
|  | Agree/Strongly agree  Neutral  Disagree/Strongly disagree | 19 (90)  0  2 (10) | -  -  - | -  -  - |
|  | Increased my confidence to improve my diet |  |  |  |
|  | Agree/Strongly agree   Neutral  Disagree/Strongly disagree | 18 (86)  1 (5)  2 (9) | -  -  - | -  -  - |
|  | Helped me to achieve my goals |  |  |  |
|  | Agree/Strongly agree   Neutral  Disagree/Strongly disagree | 17 (81)  2 (9)  2 (9) | -  -  - | -  -  - |
|  | Made me feel accountable |  |  |  |
|  | Agree/Strongly agree   Neutral  Disagree/Strongly disagree | 18 (86)  2 (9)  1 (5) | -  -  - | -  -  - |
|  | Strategies addressed barriers to healthy eating |  |  |  |
|  | Agree/Strongly agree   Neutral  Disagree/Strongly disagree | 18 (86)  1 (5)  1 (5) | -  -  - | -  -  - |
|  | Email summary of goals and strategies was useful |  |  |  |
|  | Agree/Strongly agree   Neutral  Disagree/Strongly disagree | 18 (86)  2 (9)  1 (5) | -  -  - | -  -  - |
|  | Video coaching sessions were useful |  |  |  |
|  | Agree/Strongly agree  Neutral  Disagree/Strongly disagree | 19 (90)  0  2 (10) | -  -  - | -  -  - |
|  | Video coaching was easier than attending in person |  |  |  |
|  | Agree/Strongly agree  Neutral  Disagree/Strongly disagree | 20 (95)  1 (5)  0 | -  -  - | -  -  - |
|  | Number of sessions |  |  |  |
|  | Just right  Would have preferred more contact | 13 (62)  7 (33) | -  - | -  - |
|  | Would have preferred less contact | 1 (5) | - | - |
|  | Duration of sessions |  |  |  |
|  | About right | 21 (100) | - | - |
| **Video coaching sessions: Exercise physiologist**^c^ | Provided me with useful information about exercise |  |  |  |
|  | Strongly agree  Agree  Neutral | 20 (95)  1 (5)  0 | -  -  - | -  -  - |
|  | Increased my confidence to improve my physical activity behaviours |  |  |  |
|  | Agree/Strongly agree  Neutral  Disagree/Strongly disagree | 21 (100) | - | - |
|  |  | 0  0 | -  - | -  - |
|  | Helped me to achieve my goals |  |  |  |
|  | Agree/Strongly agree  Neutral  Disagree/Strongly disagree | 18 (86) | - | -  -  - |
|  |  | 2 (9)  1 (5) | -  - |  |
|  | Motivated me to be more active |  |  |  |
|  | Agree/Strongly agree  Neutral  Disagree/Strongly disagree | 20 (95)  0  1 (5) | -  -  - | -  -  - |
|  | Made me feel accountable |  |  |  |
|  | Agree/Strongly agree  Neutral  Disagree/Strongly disagree | 20 (95) | - | - |
|  |  | 1 (5)  0 | -  - | -  - |
|  | Addressed barriers preventing me from being active |  |  |  |
|  | Agree/Strongly agree  Neutral  Disagree/Strongly disagree | 17 (81) | - | - |
|  |  | 3 (14)  1 (5) | -  - | -  - |
|  | Video coaching sessions were useful |  |  |  |
|  | Agree/Strongly agree  Neutral  Disagree/Strongly disagree | 19 (90)  2 (10)  0 | -  -  - | -  -  - |
|  | Video coaching was easier than attending in person |  |  |  |
|  | Agree/Strongly agree  Neutral  Disagree/Strongly disagree | 20 (95) | - | - |
|  |  | 1 (5)  0 | -  - | -  - |
|  | Number of sessions |  |  |  |
|  | About right | 15 (71) | - | - |
|  | Would have preferred more contact  Would have preferred less contact | 5 (24)  1 (5) | -  - | -  - |
|  | Duration of sessions |  |  |  |
|  | About right | 21 (100) | - | - |
| **Video coaching sessions: Overall**^c^ | Scheduling was appropriate |  |  |  |
|  | Agree/Strongly agree  Neutral  Disagree/Strongly disagree | 14 (67)  3 (14)  4 (19) | -  -  - | -  -  - |
|  | Picture quality was acceptable |  |  |  |
|  | Agree/Strongly agree  Neutral  Disagree/Strongly disagree | 18 (86)  2 (10)  1 (5) | -  -  - | -  -  - |
|  | Sound quality was acceptable |  |  |  |
|  | Agree/Strongly agree  Neutral  Disagree/Strongly disagree | 20 (95)  0  1 (5) | -  -  - | -  -  - |
|  | Video program was easy to use |  |  |  |
|  | Agree/Strongly agree  Neutral  Disagree/Strongly disagree | 19 (90)  1 (5)  1 (5) | -  -  - | -  -  - |
|  | Technology issues delayed/prevented me from completing a session |  |  |  |
|  | Yes | 7 (33) | - | -  - |
|  | No | 14 (67) | - |  |
|  | Level of comfort interacting via video |  |  |  |
|  | Comfortable/Very Comfortable  Neutral | 20 (95)  1 (5) | -  - | -  - |
|  | Uncomfortable/Very uncomfortable | 0 | - | - |
|  | Overall satisfaction with video coaching |  |  |  |
|  | Satisfied/Very satisfied | 16 (76) | - | - |
|  | Neutral | 2 (10) | - | - |
|  | Unsatisfied/Very unsatisfied | 3 (14) | - | - |
| **Text message support**^d^ | Provided me with useful information |  |  |  |
|  | Agree/Strongly agree  Neutral  Disagree/Strongly disagree | 17 (81) | 7 (44) | - |
|  |  | 3 (14) | 2 (13) | - |
|  |  | 1 (5) | 7 (44) | - |
|  | Made me feel accountable |  |  |  |
|  | Agree/Strongly agree | 16 (76) | 5 (31) | - |
|  | Neutral | 3 (14) | 4 (25) | - |
|  | Disagree/Strongly disagree | 2 (10) | 7 (44) | - |
|  | Increased my confidence to improve my diet and eating behaviours |  |  |  |
|  | Agree/Strongly agree | 15 (71) | 4 (25) | - |
|  | Neutral | 3 (14) | 5 (31) | - |
|  | Disagree/Strongly disagree | 3 (14) | 7 (44) | - |
|  | Increased my confidence to improve my activity level |  |  |  |
|  | Agree/Strongly agree | 13 (62) | 3 (19) | - |
|  | Neutral | 5 (24) | 6 (38) | - |
|  | Disagree/Strongly disagree | 3 (14) | 7 (44) | - |
|  | Helped me achieve my weight goals |  |  |  |
|  | Agree/Strongly agree | 11 (53) | 1 (6) | - |
|  | Neutral | 6 (29) | 6 (38) | - |
|  | Disagree/Strongly disagree | 4 (19) | 9 (56) | - |
|  | Helped me achieve my nutrition goals |  |  |  |
|  | Agree/Strongly agree | 11 (52) | 3 (19) | - |
|  | Neutral | 6 (29) | 5 (31) | - |
|  | Disagree/Strongly disagree | 4 (19) | 8 (50) | - |
|  | Helped me achieve my exercise goals |  |  |  |
|  | Agree/Strongly agree | 10 (48) | 2 (13) | - |
|  | Neutral | 7 (33) | 6 (38) | - |
|  | Disagree/Strongly disagree | 4 (19) | 8 (50) | - |
|  | Helped me eat more healthily |  |  |  |
|  | Agree/Strongly agree | 14 (67) | 2 (13) | - |
|  | Neutral | 3 (14) | 6 (38) | - |
|  | Disagree/Strongly disagree | 4 (19) | 4 (25) | - |
|  | Addressed healthy eating barriers |  |  |  |
|  | Agree/Strongly agree | 12 (57) | 3 (19) | - |
|  | Neutral | 5 (24) | 3 (19) | - |
|  | Disagree/Strongly disagree | 1 (5) | 10 (62) | - |
|  | Helped me be more active |  |  |  |
|  | Agree/Strongly agree | 9 (43) | 1 (6) | - |
|  | Neutral | 9 (43) | 7 (44) | - |
|  | Disagree/Strongly disagree | 3 (14) | 8 (50) | - |
|  | Addressed exercise barriers |  |  |  |
|  | Agree/Strongly agree | 9 (43) | 2 (13) | - |
|  | Neutral | 8 (38) | 4 (25) | - |
|  | Disagree/Strongly disagree | 4 (19) | 10 (62) | - |
|  | Useful reminder to reflect on weight goals |  |  |  |
|  | Agree/Strongly agree | 14 (67) | 5 (31) | - |
|  | Neutral | 5 (24) | 3 (19) | - |
|  | Disagree/Strongly disagree | 2 (10) | 8 (50) | - |
|  | Useful reminder to reflect on nutrition goals  Agree/Strongly agree  Neutral  Disagree/Strongly disagree | 15 (75)  4 (19)  2 (10) | 5 (31)  3 (19)  8 (50) | -  -  - |
|  | Useful reminder to reflect on exercise goals  Agree/Strongly agree  Neutral  Disagree/Strongly disagree |  |  |  |
|  |  | 15 (75)  4 (19)  1 (5) | 3 (19)  4 (25)  9 (56) | -  -  - |
|  | Useful reminder to self-monitor  Agree/Strongly agree  Neutral  Disagree/Strongly disagree |  |  |  |
|  |  | 14 (67)  5 (24)  2 (10) | 5 (31)  3 (19)  8 (50) | -  -  - |
|  | Personalised to my healthy eating barriers  Agree/Strongly agree  Neutral  Disagree/Strongly disagree |  |  |  |
|  |  | 12 (57)  7 (33)  2 (10) | 5 (31)  5 (31)  6 (38) | -  -  - |
|  | Personalised to my exercise barriers |  |  |  |
|  | Agree/Strongly agree  Neutral  Disagree/Strongly disagree | 13 (62)  6 (29) | 3 (19)  7 (44) | - |
|  |  | 2 (10) | 6 (38) | - |
|  | Number of text messages |  |  |  |
|  | Just right  Would have preferred more  Would have preferred less | 11 (52) | 6 (38) | - |
|  |  | 5 (24) | 5 (31) | - |
|  |  | 5 (24) | 5 (31) | - |
|  | Overall satisfaction with text messages |  |  |  |
|  | Satisfied/Very satisfied  Neutral  Unsatisfied/Very unsatisfied | 15 (71) | 6 (38) | - |
|  |  | 3 (14) | 3 (19) | - |
|  |  | 3 (14) | 7 (44) | - |
| **Body Balance Beyond: Overall program** | I would recommend the program to other women with GDM |  |  |  |
|  | Agree/Strongly agree  Neutral  Disagree/Strongly disagree | 18 (86) | 7 (44) | 5 (36) |
|  |  | 2 (10)  1 (5) | 4 (25)  5 (31) | 4 (29)  5 (36) |
|  | The program met my expectations |  |  |  |
|  | Agree/Strongly agree | 15 (71) | 4 (25) | 2 (14) |
|  | Neutral | 2 (10) | 3 (19) | 5 (36) |
|  | Disagree/Strongly disagree | 4 (19) | 9 (56) | 7 (50) |
|  | I was satisfied with the program |  |  |  |
|  | Agree/Strongly agree | 14 (67) | 4 (25) | 2 (14) |
|  | Neutral | 4 (19) | 3 (19) | 5 (36) |
|  | Disagree/Strongly disagree | 3 (14) | 9 (56) | 7 (50) |

^a^*n=*53 participants completed the study, process evaluation data missing for *n*=2 participants.

^b^ Responses shown for participants who responded ‘Yes’ to accessing the relevant website component at the start of each section.

^c^ High Personalisation group only

^d^ High and Medium Personalisation groups only

Abbreviations: BBB, Body Balance Beyond; GDM, gestational diabetes mellitus; HP, High Personalisation; MP, Medium Personalisation; LP, Low Personalisation.

**Results**

### Acceptability

Results from the process evaluation survey at 3 months for the HP, MP and LP groups are provided in Additional file 3. Survey responses related to the intervention components received by all 3 groups (AES and physical activity reports, and the ‘Body Balance Beyond’ website) were not analysed statistically due to the small sample sizes [HP (n=21), MP (n=16), LP (n=14), missing data (n=2)].

*Australian Eating Survey and Physical Activity reports (all groups)*

The majority of women read or saw the AES report (n=41, 77%) while fewer (22%) read or saw the Physical Activity (n=29, 55%) report. The percentage of women who accessed these reports was higher in the HP group (91%) compared to the MP (69%) and LP (79%). Many women found the AES and Physical Activity reports easy to understand (n=32, 78% and n=27, 93%, respectively). Feedback from these reports helped many women identify areas for improvement of their dietary intake (n=35, 85%) and physical activity levels (n=29, 100%).

*‘Body Balance Beyond’ website (all groups)*

Many women (n=40, 75%) accessed the ‘Body Balance Beyond’ website and felt it provided useful information about diabetes risk (n=29, 73%). This perception was higher in the HP group (82%) compared to the MP (69%) and LP (60%) groups. Only a quarter of women found the website motivating for modifying their health behaviours, although an equal proportion of women felt more accountable for their health behaviour. Of the women who accessed the website, 58% used the goal setting module at least once per month. Some women agreed that the module assisted with setting weight (n=17, 43%), nutrition (n=19, 48%) and exercise (n=16, 40%) goals. Over half the sample of women were satisfied with how the website supported them in managing their diabetes risk (n=23, 58%) while fewer were satisfied with how the website supported them in developing a plan (n=19, 40%).

*Text message support (HP and MP groups only)*

Some women (n=24, 65%) agreed that the text messages provided useful information. For instance, one participant indicated that text messages “were a great reminder to aim to stay focused and to not fall backwards into bad habits.” Approximately half of the women agreed that text messages increased their confidence to improve their diet (n=19, 51%) and physical activity levels (n=19, 51%). Only one third of women agreed that text messages helped them to achieve their weight (n=12, 32%), nutrition (n=14, 38%) and exercise goals (n=12, 32%). Some women (n=10, 27%) would have preferred to have received more text messages. Overall, just over half the women (n=21, 57%) were satisfied with the text messages provided, and satisfaction was higher in the HP group (71%) compared to the MP group (38%).

*Video coaching sessions (HP group only)*

Twenty-one women (100%) from the HP group responded to survey questions related to the video coaching sessions. Of these women, most agreed that the video coaching sessions with the dietitian and the exercise physiologist provided useful information (n=19, 90% and n=21, 100%, respectively). Most women agreed that these sessions increased their confidence with improving their diet and physical activity levels (n=18, 86% and n=21, 100%). Some women would have preferred to have more video coaching sessions with the dietitian (n=7, 33%) or exercise physiologist (n=5, 24%). All women (100%) were satisfied with the duration time for the sessions (20-30 minutes). One third (33%) of women experienced technological issues that delayed or prevented them completing a session. Most women (95%) still agreed that video coaching sessions were easier to attend than in-person sessions. Overall, a high proportion (76%) of women were satisfied with the video coaching sessions.
