# Supplementary material for: Evaluation of a Type 2 diabetes risk reduction online program for women with recent gestational diabetes: a randomised trial
Source: Int J Behav Nutr Phys Act. 2022 Mar 28;19:35. doi: 10.1186/s12966-022-01275-3 (PMC8962548; doi:10.1186/s12966-022-01275-3)
Supplement: Supplementary file 4 — Additional file 4: Supplementary file 2. Results for Exercise Self-Efficacy Scale model including two-way interaction of age x time. [file 12966_2022_1275_MOESM4_ESM.docx]

**Supplementary file 2.** Results for Exercise Self-Efficacy Scale model including two-way interaction of age x time.

| **Outcome** | **HP**  **Group**  n = 25 | **MP**  **Group**  n = 23 | **LP**  **Group**  n = 28 | **MP vs LP groups** | **HP vs LP groups** | **HP vs MP groups** | **P-value** |
| --- | --- | --- | --- | --- | --- | --- | --- |
| Exercise Self-Efficacy Scale^a^  (max. 90) | 5.37  (4.92, 5.83) | -5.54  (-6.53, -4.55) | -3.28  (-4.81, -1.75) | -1.34  (-13.34, 10.66) | 9.37  (-1.97, 20.71) | 10.71  (-0.73, 22.15) | .11 |

^a^ Adjusted for age and two-way interaction of age x time.
